# Supplementary material for: Single-cell sequencing of tumor-associated macrophages in a Drosophila model
Source: Front Immunol. 2023 Sep 19;14:1243797. doi: 10.3389/fimmu.2023.1243797 (PMC10546068; doi:10.3389/fimmu.2023.1243797)
Supplement: Supplementary file 1 [file DataSheet_1.docx]

Supplementary Material

Single-cell sequencing of tumor-associated macrophages in a *Drosophila* model

Dilan Khalili^#1^, Mubasher Mohammed^#1^, Martin Kunc^#1, 2^, Martina Sindlerova^1^, Johan Ankarklev^1^, Ulrich Theopold^1*^

*** Correspondence:** Ulrich Theopold: uli.theopold@su.se

**^#^equal contribution**

#
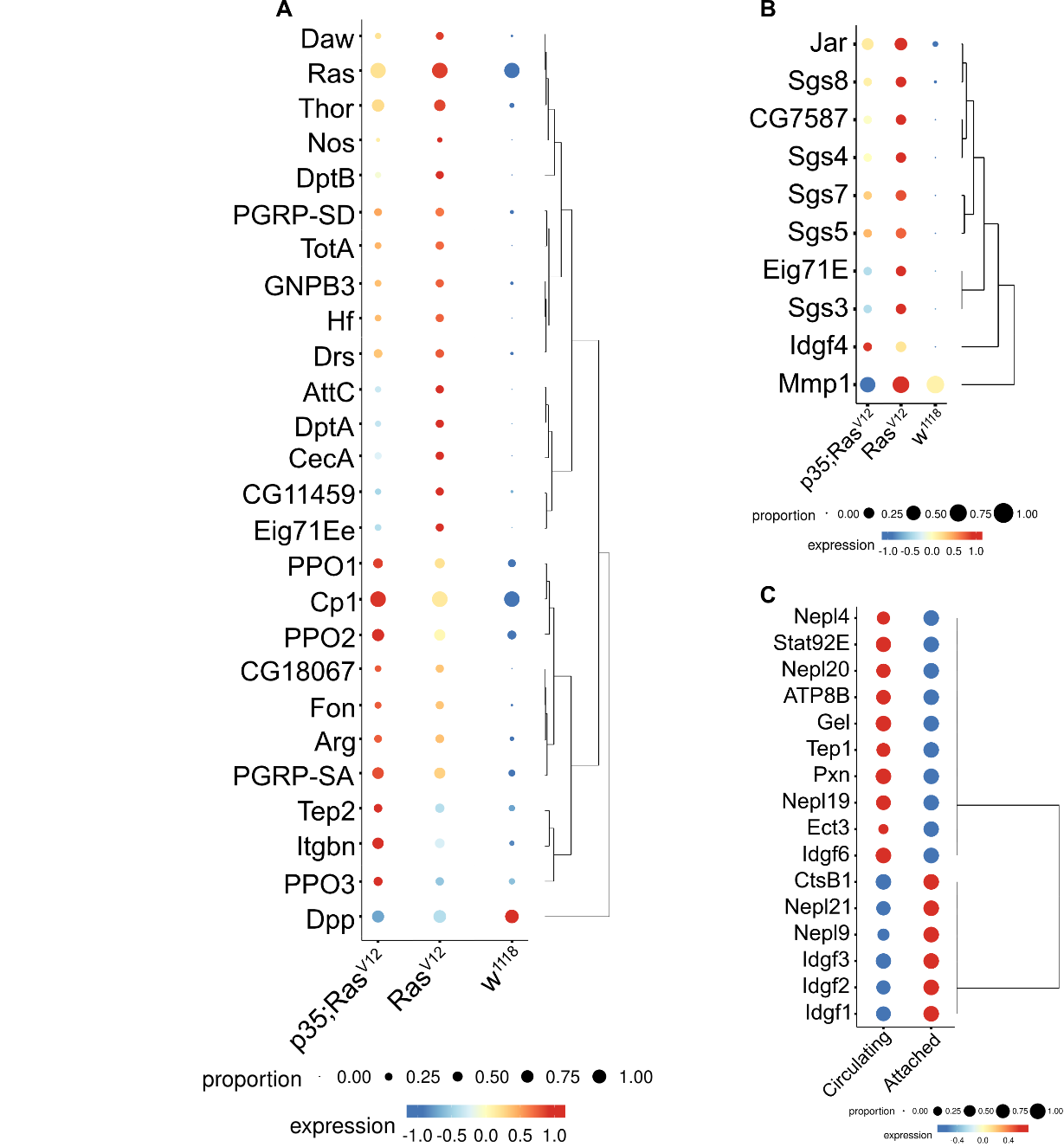
Supplementary Figures

**Figure S1**

Split analysis for the genes in cluster 2 using the category Biological function which identified the terms **(A)** “Immune system process” and **(B)** “Molting process” as most significant. These two terms were compared between the three genotypes. **(C)** Genotype-specific analysis for the genes in cluster 0 in the Reactome pathway “Neutrophil degranulation” were compared between attached vs circulating cells.


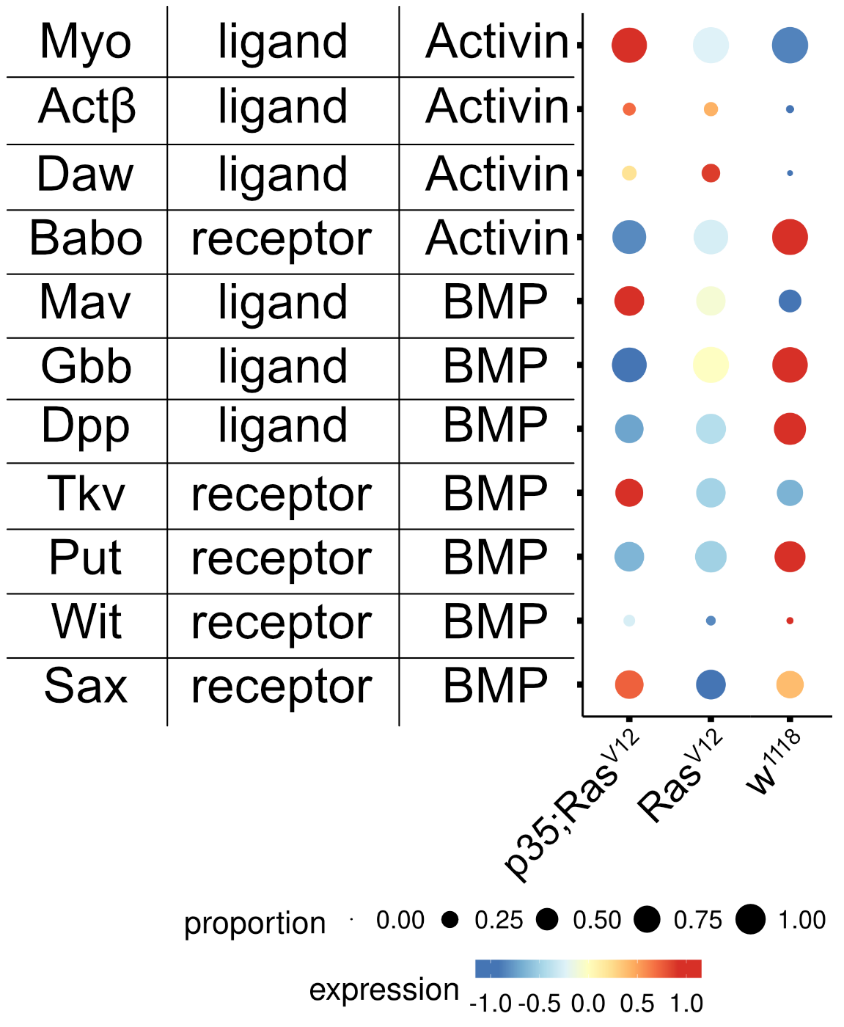


**Figure S2**

Split analysis for the genes involved in tumor necrosis factor (TGF) signaling using https://mubashe r-mohammed.shinyapps.io/Sc-drosophila/?_ga=2.6566386.119719391.1657634466-1490444630.1643316020. Both members of the activin- and BMP-like pathway are shown including ligands and receptors.


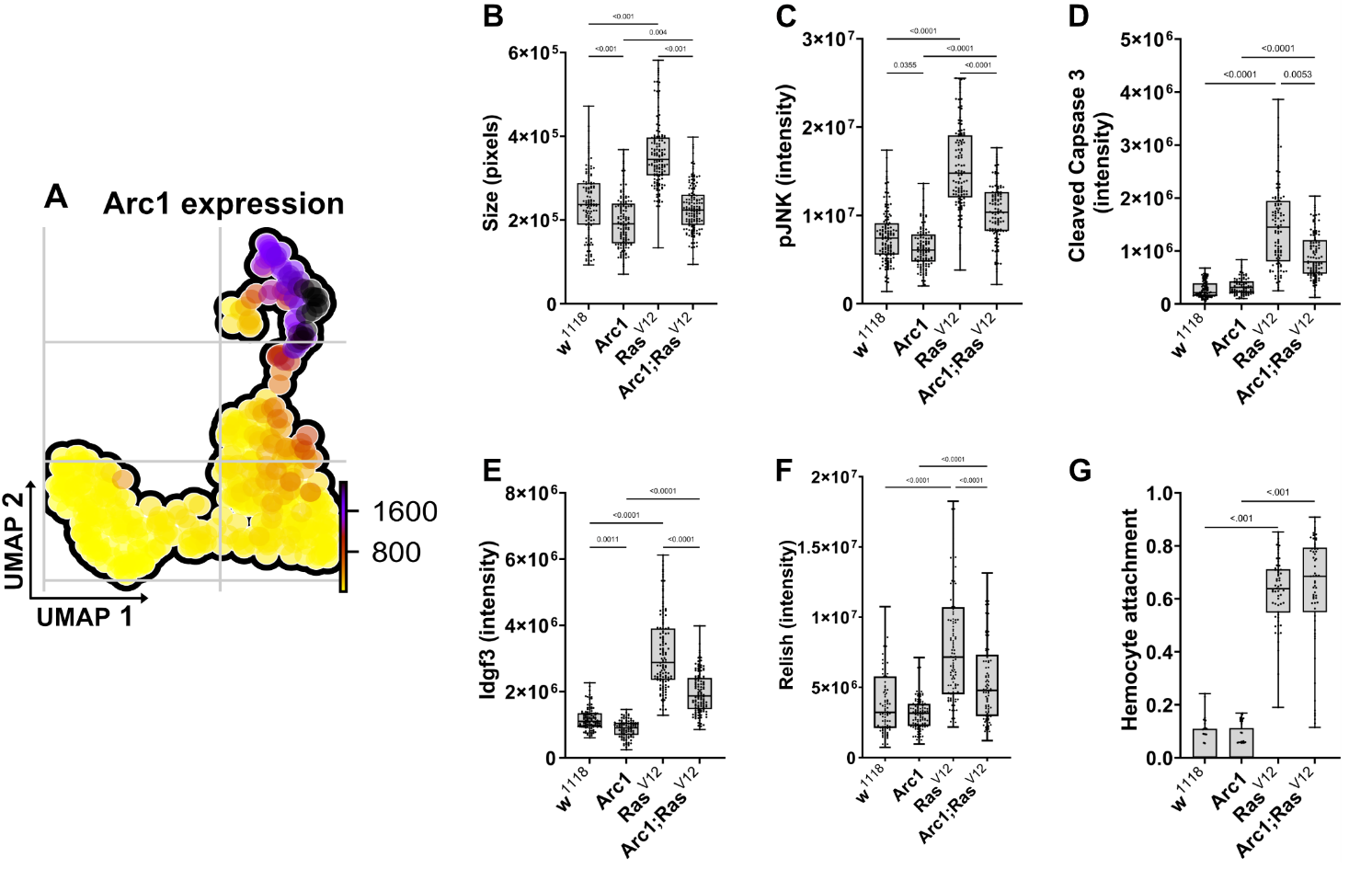


**Figure S3**

**Characterization of homozygous Arc1 mutant**

**(A)** UMAP visualizes transcripts analysis showing the strongest expression of Arc1 gene in TAHs. Quantification of size **(B),** expression of pJNK **(C),** Cleaved Caspase 3 **(D),** Idgf3 **(E)**, Relish **(F)** and Hemocyte attachment **(G)** show alleviation of some Ras-associated phenotypes**.** Whisker length min to max, bar represent median. P-value quantified with ANOVA (antibody in F: anti-Relish-C 21F3-s; (1:100, Developmental Studies Hybridoma Bank).

.
